# Evaluation of Crack Formation in Heat Pipe-Welded Joints

**DOI:** 10.3390/ma18092028

**Published:** 2025-04-29

**Authors:** Min Ji Song, Keun Hyung Lee, Jun-Seob Lee, Heesan Kim, Woo Cheol Kim, Soo Yeol Lee

**Affiliations:** 1 Department of Materials Science and Engineering, Chungnam National University, Daejeon 34134, Republic of Korea; minji0722@o.cnu.ac.kr (M.J.S.); porary95@o.cnu.ac.kr (K.H.L.); 2 School of Materials Science and Engineering, Changwon National University, Changwon-si 51140, Republic of Korea; junseoblee@changwon.ac.kr; 3 Department of Nanomaterials, Hongik University, Sejong-si 30016, Republic of Korea; hskim@hongik.ac.kr; 4 Plant Management & QC Division, Korea District Heating Corp., Seongnam-si 13585, Republic of Korea; kwc7777@kdhc.co.kr

**Keywords:** district heating system, galvanic corrosion, dual-insulated pipeline, weld failure

## Abstract

This study investigates the failure of a 750A dual-insulated pipeline, where cracks developed along the weld joints during heat supply resumption at the district heating facility. A comprehensive analysis was conducted through visual inspection, mechanical testing, microstructural characterization, finite element analysis (FEA), and electrochemical corrosion testing. The results indicate that cracks were generated in the heat-affected zone (HAZ), primarily caused by galvanic corrosion and thermal expansion-induced stress accumulation. Open circuit potential (OCP) measurements in a 3 M NaCl solution confirmed that the HAZ was anodic, leading to the most vulnerable position to corrosion. Furthermore, localized electrochemical tests were conducted for respective microstructural regions within the HAZ. The results reveal that coarse-grained HAZ exhibited the lowest corrosion potential, giving rise to preferential corrosion, promoting pit formation, and serving as initiation sites for stress concentration and crack propagation. FEA simulations demonstrate that pre-existing microvoids in the HAZ act as stress concentration sites, undergoing a localized stress exceeding 475 MPa. These findings emphasize the importance of controlling microstructural stability and mechanical integrity in welded pipelines, particularly in corrosive environments subjected to thermal stresses.

## 1. Introduction

District heating pipelines are critical infrastructure for the distribution of thermal energy in urban networks. These pipelines are often buried underground, exposing them to harsh environmental conditions that accelerate material degradation over time. Factors such as cyclic thermal fluctuations, internal pressure variations, and chemically aggressive environments contribute to the progressive deterioration of pipeline materials, raising concerns about their long-term structural integrity [1]. Among various degradation mechanisms, corrosion and mechanical fatigue are particularly problematic in underground district heating systems.

Corrosion in buried pipelines manifests in different forms depending on environmental and operational conditions. Pitting and crevice corrosion frequently occur at welds and inner pipe surfaces due to differential oxygen concentrations and moisture accumulation, leading to localized material loss and structural weakening [2,3,4,5]. Additionally, the type of fluid transported within pipelines significantly influences corrosion behavior. For instance, pipelines carrying landfill gas or biogas are particularly susceptible to hydrogen sulfide (H_2_S)-induced corrosion, leading to sulfide stress cracking (SSC) and metal embrittlement [6,7,8]. In contrast, pipelines transporting steam or high-temperature water often experience accelerated oxidation and flow-assisted corrosion (FAC), exacerbating wall thinning and structural degradation [9,10,11]. Given these degradation mechanisms, welded joints have been identified as critical weak points in district heating and high-temperature pipeline systems. Extensive research on welded pipeline failures has shown that degradation mechanisms vary depending on environmental and operational factors. The heat-affected zone (HAZ), in particular, is susceptible to failure due to microstructural modifications, residual stress accumulation, and electrochemical instability induced by thermal cycling [12,13]. Studies on pipelines used in diverse environments, including oil transport, fire protection, and district heating systems, have consistently demonstrated the vulnerability of welded joints to cracking, galvanic corrosion, and fatigue-related damage under cyclic stress conditions, highlighting weld defects, residual stress, and metallurgical instability as key contributors to premature failure [14,15,16,17,18]. While these studies provide valuable insights, further investigation is needed to understand the interplay between corrosion, mechanical stress, and thermal cycling in district heating pipelines, particularly those buried underground where unique environmental conditions affect material degradation.

This study examines the failure of a 750A dual-insulated district heating pipeline, where cracking was detected along welded joints following the resumption of heat supply after a temporary shutdown. To verify this, a comprehensive investigation was conducted by integrating visual inspection, mechanical property evaluation, microstructural analysis, finite element stress simulation, and electrochemical corrosion testing. Particular emphasis was placed on quantifying galvanic potential distributions across the weldment, assessing the impact of thermal stress on crack propagation, and correlating microstructural changes in the HAZ with failure patterns. The findings of this study aim to improve corrosion mitigation strategies, optimize welding procedures, and enhance predictive maintenance methodologies, ultimately contributing to the long-term reliability of district heating pipeline systems.

## 2. Materials and Methods

**Figure 1:**
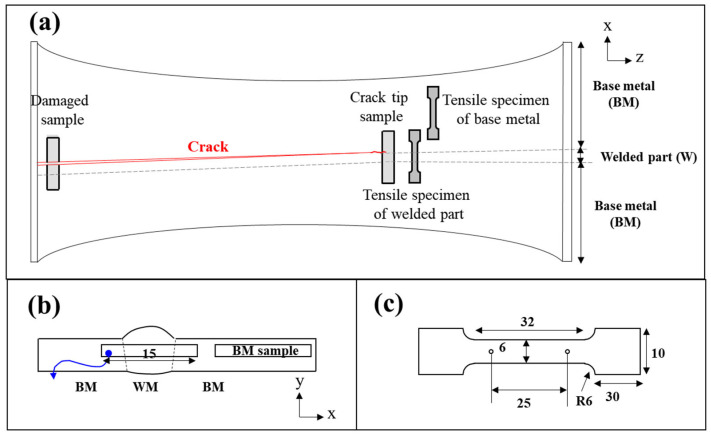
Schematics of the pipe sample and specimen extraction locations: (**a**) crack locations and specimen positions, (**b**) specimens for microstructural and electrochemical analysis, and (**c**) geometry of the tensile test specimen.

The pipeline was fabricated using an inert gas shielded arc welding process, either gas tungsten arc welding (GTAW, TIG) or gas metal arc welding (GMAW, MIG), which are commonly applied for carbon steel pipe joints in industrial practice. Dual-insulated pipe samples were collected from the field using arc cutting to investigate the failure mechanisms of the district heating pipeline (Figure 1 and Table 1). A visual inspection was initially performed to roughly assess the crack morphology and locations, followed by the processing of precise samples for further analysis. Subsequent experimental analyses included mechanical property evaluation, microstructural characterization, stress analysis, and electrochemical testing.

For mechanical property evaluation, tensile specimens were prepared to include both weld metal and base metal within the gauge length, while additional reference specimens were extracted from a region sufficiently away from the weld line to eliminate welding effects (Figure 1a). Tensile tests were conducted using a universal testing machine at a strain rate of 10^−3^ s^−1^. Hardness mapping was performed using the Vickers hardness test with a 0.3 kgf test load, with measurements taken at 0.3 mm intervals in both the x- and y-directions to analyze localized variations. This spacing was selected to satisfy the commonly recommended rule of maintaining a distance of at least three indentation diameters between adjacent impressions.

Microstructural characterization was carried out on specimens categorized into cracked and uncracked regions, including areas near the crack tip and sections without observable cracks. Cross-sectional microstructures were examined using optical microscopy after etching with a 5% Nital solution. Additionally, fracture surface analysis was performed using scanning electron microscopy (SEM) (Carl Zeiss, Oberkochen, Germany) after descaling to investigate crack propagation behavior. Additionally, electron backscatter diffraction (EBSD) analysis was conducted to quantify grain morphology and misorientation distribution. Grain size data were obtained using the TSL OIM Analysis™ software (version 7.0), where grain boundaries were defined using a 5° misorientation threshold. The average grain size was calculated by the equivalent circle diameter method, in which the area of each grain is converted into the diameter of a circle with the same area.

A stress analysis simulation was performed using ANSYS software (2022 R2) to assess stress concentration and crack formation potential. A 2D model of an uncracked sample cross-section was reconstructed in CAD based on optical microscopy (OM) images. The thermal stress distribution was calculated using supply temperature history (°C/min) and tensile test data. The pipe’s outer surface was assumed to be fixed due to constraints imposed by HDPE and polyurethane foam insulation, while external factors, such as soil pressure and vibrations, were neglected for simplification.

Electrochemical characterization was conducted to evaluate the corrosion behaviors of the base metal, weld metal, and HAZ. Open circuit potential (OCP) measurements were performed in a 3 M NaCl solution using specimens extracted along the longitudinal direction, selected based on hardness test results and microstructural observations. Weld and HAZ specimens were obtained from the same region and further distinguished through microstructural analysis and surface treatment. Additionally, localized electrochemical testing was carried out to assess the corrosion susceptibility of specific microstructural regions. These tests were performed in a 50 mM NaCl solution, targeting selected areas to evaluate microstructural influences on corrosion behavior under lower ionic strength conditions. The electrochemical setup included an amorphous carbon (CE) counter electrode, an Ag/AgCl (saturated KCl) reference electrode, a scan rate of 0.5 mV/s, and a potential range from −0.1 Vocp to Vsse at 500 µA/cm^2^.

## 3. Results

### 3.1. Visual Inspection and Thickness Measurement

**Figure 2:**
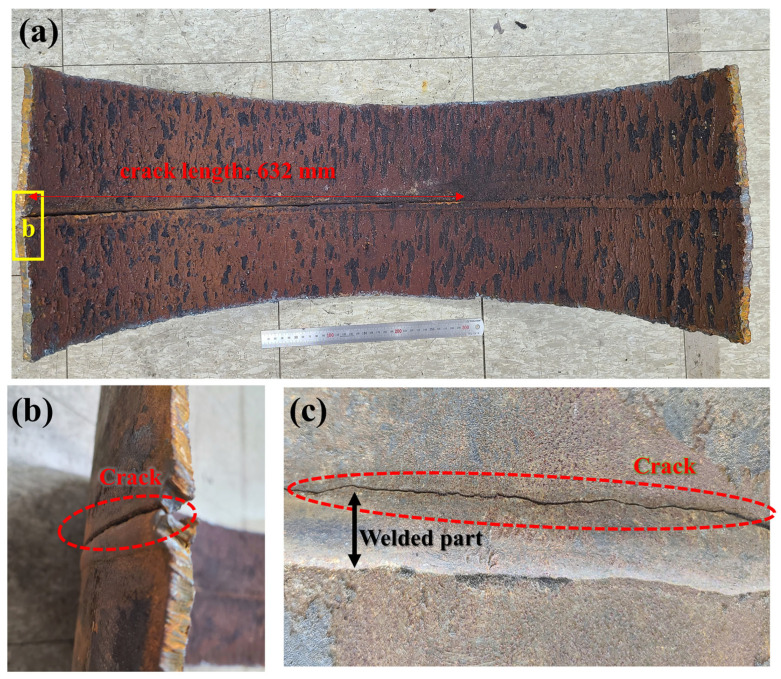
Visual inspection of pipe sample: (**a**) pipe picture, (**b**) crack (outside), and (**c**) crack on weld toe.

The visual inspection of the collected pipe specimens revealed distinct crack propagation along the circumferentially welded seam, with cracks predominantly initiating and extending along the weld toe (Figure 2). The crack morphology indicates that failure was concentrated along one side of the weld toe, following the weld bead along the pipe’s circumferential weld seam. No significant surface defects, such as pitting, structural deformation, or mechanical indentations, were observed during the visual examination. However, localized stress concentration along the weld region was identified as a potential initiator of crack formation. The combination of residual stress from welding and operational loading is inferred to have accelerated crack propagation in the absence of widespread material degradation.

### 3.2. Mechanical Properties

**Figure 3:**
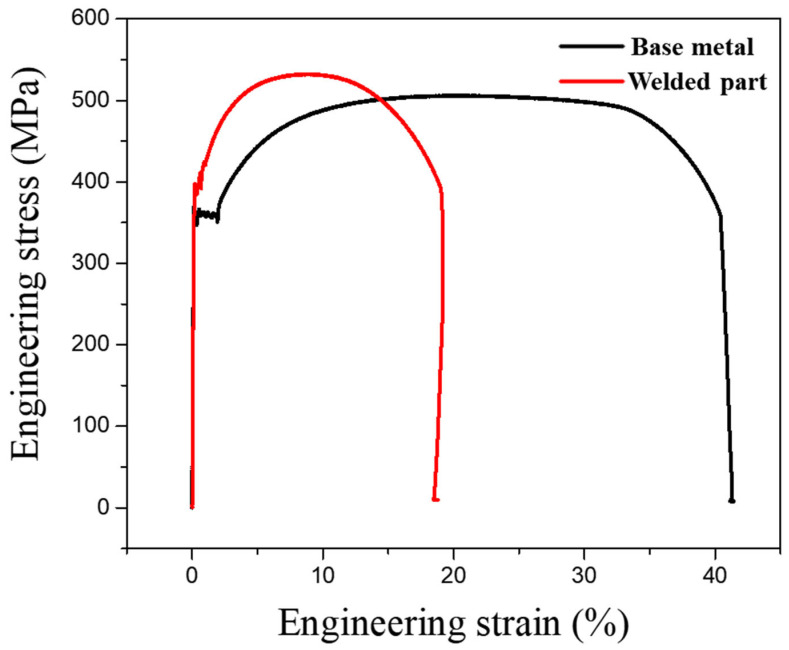
Tensile test result curve of base metal and welded part.

Tensile test results revealed distinct variations in the mechanical properties of the base metal, weld metal, and HAZ (Figure 3). The base metal exhibited superior ductility, with a strain-to-failure of 41.2%, whereas the weld metal demonstrated a significantly lower elongation of 18.7%, indicating reduced deformability. The ultimate tensile strength (UTS) of the weld metal was measured at 532 MPa, slightly exceeding that of the base metal (506 MPa). Despite the marginally higher UTS, the reduced elongation in the weld region suggests increased brittleness tendency, likely due to microstructural transformations induced by welding. These findings indicate that welding alters the mechanical behavior of the material, particularly affecting its ductility and failure characteristics. A summary of the tensile test results is presented in Table 2.

**Figure 4:**
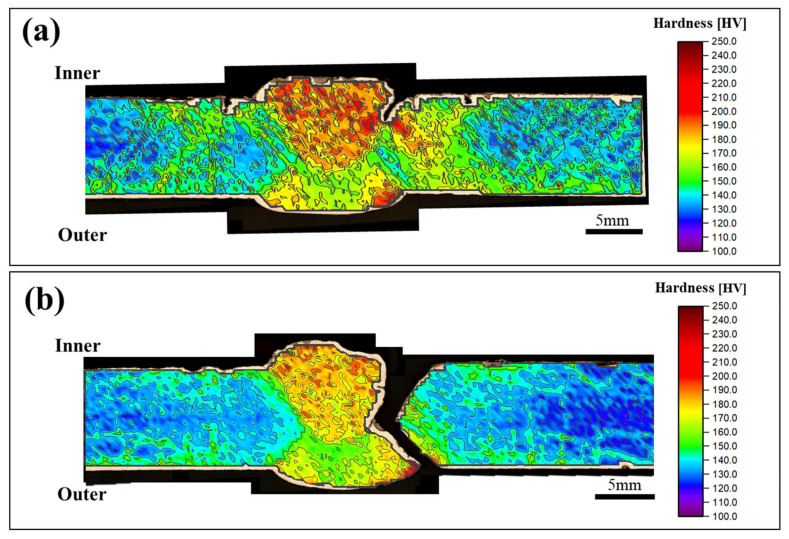
Hardness test result: (**a**) cracked sample and (**b**) completely-fractured sample.

As shown in Figure 4, hardness mapping showed a clear gradient from the base metal to the weld metal, with the highest values found in the coarse-grained heat-affected zone (CGHAZ). The maximum hardness in this region exceeded 300 HV and gradually decreased toward the base metal, where values stabilized around 180 HV. This variation is attributed to differences in microstructural characteristics across the welded joint. In the coarse-grained zone, thermal exposure led to grain coarsening and the possible formation of harder transformation products, contributing to increased hardness and reduced ductility. In contrast, the base metal exhibited a relatively uniform ferrite and pearlite structure, resulting in lower hardness and improved deformability. The increased hardness in the heat-affected zone correlates with the observed crack propagation path, suggesting that this region is more susceptible to failure due to its reduced toughness and elevated residual stresses [12,13]. Hardness comparisons between cracked and uncracked samples further confirmed that cracks mostly originated from the coarse-grained region, where both structural instability and localized stress were present.

The fractured and unfractured specimens were both taken from the same weld line and are considered to have undergone similar welding conditions. Interestingly, the unfractured specimen exhibited slightly higher hardness at the weld joint, yet no failure was observed. This indicates that hardness alone is not sufficient to predict fracture behavior. Instead, local variations in residual stress, microstructural heterogeneity, or the presence of pre-existing defects may have contributed to the difference in fracture susceptibility. These results highlight that mechanical failure in welded joints is governed by the interplay of multiple factors, and localized conditions within a weld can significantly influence crack initiation, even under similar processing environments.

### 3.3. Microstructural Analysis

**Figure 5:**
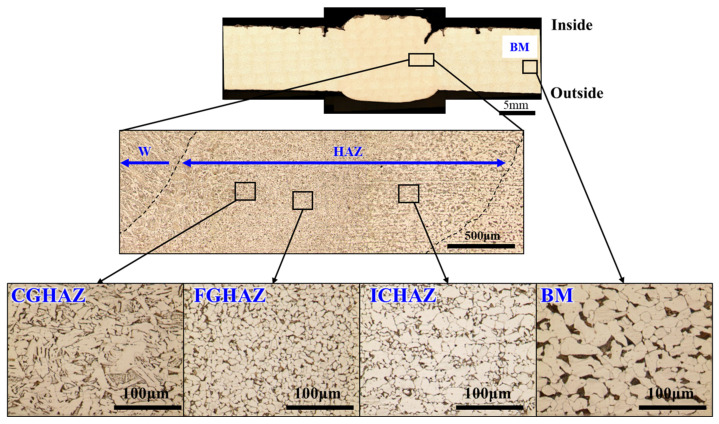
Optical micrographs of the welded joint, showing distinct microstructural regions across the base metal (BM), heat-affected zone (HAZ), and weld metal (WM).

Microstructural characterization was performed to investigate the differences in microstructures, grain morphology, and deformation characteristics across the HAZ and base metal. As shown in Figure 5, OM imaging of etched cross-sections revealed distinct microstructural transitions within the HAZ, which was subdivided into the coarse-grained HAZ (CGHAZ), fine-grained HAZ (FGHAZ), and intercritical HAZ (ICHAZ). The CGHAZ, which experienced the highest peak temperatures during welding, displayed significantly enlarged prior-austenite grains (d = 52.5 μm) with a Widmanstätten ferrite and bainitic structure, indicative of grain coarsening due to excessive thermal exposure. In contrast, the FGHAZ exhibited a finer grain structure (d = 7.6 μm) with a more uniform distribution of ferrite.

**Figure 6:**
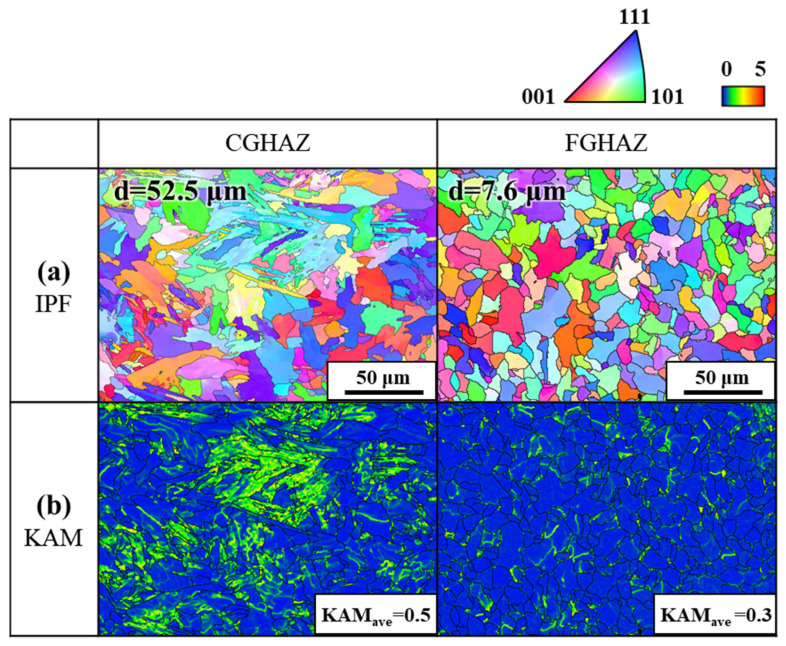
EBSD IPF and KAM maps of the analyzed microstructures: (**a**) IPF maps with average grain sizes, and (**b**) KAM maps.

Further analysis through electron backscatter diffraction (EBSD) provided quantitative insights into the grain morphology characteristics and residual strain distribution, as shown in Figure 6. The inverse pole figure (IPF) maps revealed a substantial contrast in grain morphology between CGHAZ and FGHAZ. Kernel average misorientation (KAM) mapping in Figure 6b further validated these findings. The average KAM value in CGHAZ was measured as 0.5, significantly higher than that of FGHAZ (KAM_ave_ = 0.3), indicating higher residual strain accumulation within CGHAZ.

The substructured ferritic matrix within the CGHAZ, characterized by a predominance of low-angle boundaries (LABs), contributes to strain localization and reduced fracture resistance. This region also exhibited elevated kernel average misorientation (KAM) values, which are attributed to high peak temperatures during welding followed by rapid cooling. Such thermal cycles suppress complete static recrystallization, resulting in the retention of unrecovered dislocations. Furthermore, steep thermal gradients and stress concentrations near the fusion boundary induce localized plastic deformation, leading to the accumulation of geometrically necessary dislocations (GNDs). These factors collectively contribute to the increased dislocation density observed in the CGHAZ. In contrast, the FGHAZ displayed a more homogeneous misorientation distribution and lower KAM values, indicating a relatively stabilized microstructure that experienced moderate thermal exposure and partial recovery.

**Figure 7:**
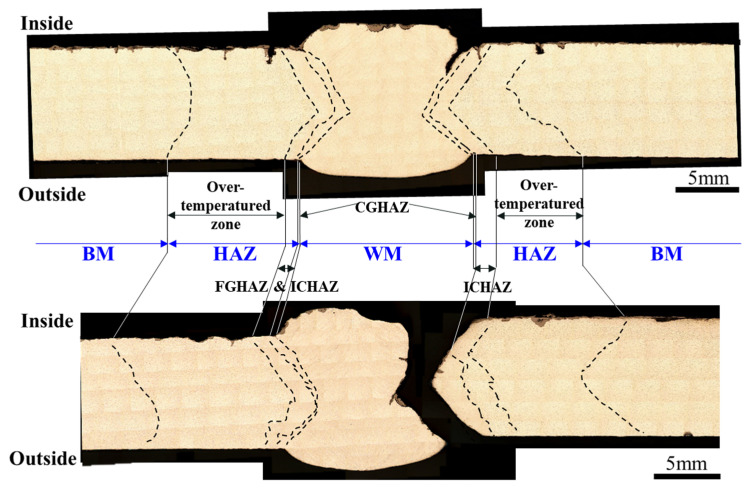
Cross-sectional comparison of fractured and unfractured welded joints, illustrating crack propagation behavior across the base metal (BM), heat-affected zone (HAZ), and weld metal (WM).

These microstructural findings correlate well with the hardness mapping results shown in Figure 7. The fracture morphology in Figure 7 indicates that crack propagation predominantly occurred in the CGHAZ, aligning with the observed high residual stress and substructured microstructure. The over-tempered zone within CGHAZ, where excessive thermal exposure led to grain coarsening and local softening, was identified as the primary fracture initiation site. These results confirm that the combination of grain coarsening, high residual stress, and low-angle boundary dominance in CGHAZ collectively contribute to its lower fracture toughness, making it the primary site for crack initiation and propagation. Furthermore, the formation of oxide layers observed on the fracture surface near the CGHAZ may also imply the presence of local chemical activity during service, suggesting that oxidative or corrosive effects may have contributed alongside mechanical degradation.

### 3.4. Stress Analysis Simulation

In order to replicate actual service conditions, a thermal boundary condition was applied to reflect the operational temperature profile. The temperature increased at a rate of 4 °C per minute, reaching 80 °C within 20 min, followed by a steady-state condition. This transient thermal gradient, combined with mechanical constraints, induced significant stress fluctuations in the HAZ and adjacent weld regions.

**Figure 8:**
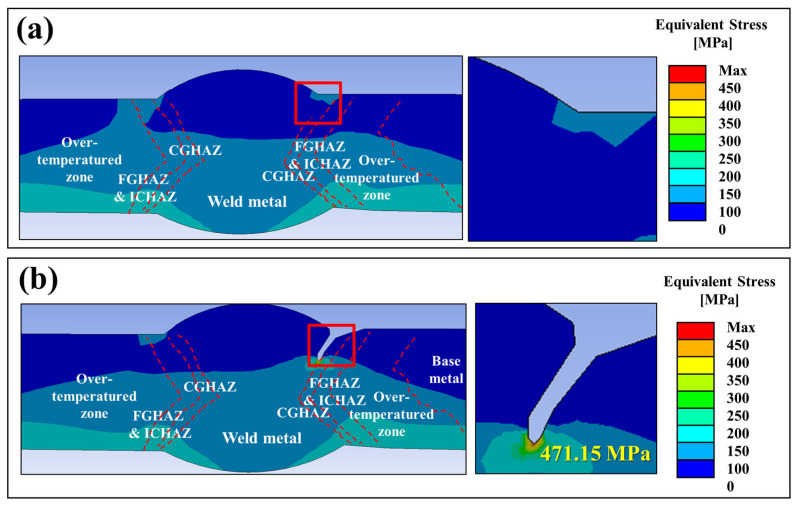
Finite element analysis (FEA) results of the welded joint. (**a**) Uncracked model showing stress distribution, (**b**) Cracked model showing stress concentration at the weld toe in the CGHAZ.

To evaluate the effect of the thermal loading on stress distribution, finite element analysis (FEA) was performed, as shown in Figure 8. The results indicated that the highest stress concentration occurred at the weld toe, particularly in the CGHAZ. The simulation incorporated both operational loading conditions and thermally induced expansion effects experienced by the pipeline during service. The maximum equivalent stress reached approximately 475 MPa, exceeding the yield strength of the base metal (349 MPa) and approaching the yield limit of the weld metal (384 MPa). Additionally, the presence of geometric discontinuities, such as weld undercut or minor misalignment, further intensified local stress concentration effects. Previous studies have shown that geometrical defects such as undercut and misalignment can act as local stress concentrators, accelerating crack initiation and propagation in welded structures, particularly under thermal and mechanical loading conditions [14,16,17]. However, considering that the observed crack propagated 632 mm along the weld toe, these results suggest that thermal expansion alone may not have been sufficient to drive such extensive fracture development. If a pre-existing crack with a sharp tip had already formed within the weld toe, the thermal expansion-induced stress could have been sufficient to trigger crack propagation, even in the absence of external mechanical overload. In such a case, the localized stress at the crack tip would have been significantly amplified, making it more susceptible to failure under the applied thermal loading.

Furthermore, it should be noted that the FEA model was constructed based on a sample extracted near the crack tip, rather than the precise crack initiation site. Therefore, the computed stress values likely underestimate the actual stress at the crack initiation location, as stress distributions typically decrease with increasing the distance from the origin. While the simulation provides valuable insights into the stress state within the HAZ, it does not fully capture the maximum stress conditions that may have existed at the precise crack initiation point.

The presence of an extremely sharp crack tip suggests that factors beyond mechanical loading may have contributed to crack initiation and propagation. Given that the weld metal and CGHAZ exhibited distinct microstructural characteristics, the possibility of a localized electrochemical gradient cannot be excluded. Such a gradient could have facilitated preferential dissolution at the weld toe, further sharpening the crack tip and reducing the mechanical stress required for propagation. Previous studies have demonstrated that the combined effects of mechanical stress and electrochemical interactions at the crack tip can accelerate stress corrosion crack growth, particularly in high-strength steels exposed to corrosive environments [19]. The role of electrochemical effects in crack initiation and propagation is further examined in the following section, where the corrosion resistance of individual microstructural regions is quantitatively evaluated through electrochemical polarization.

### 3.5. Electrochemical Properties

**Figure 9:**
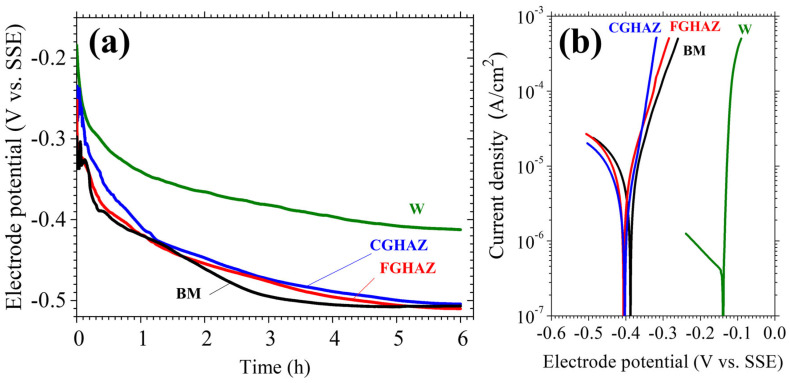
Electrochemical experiment result: (**a**) open circuit potential test and (**b**) potential dynamic curve.

To evaluate the influence of microstructural variations on electrochemical stability, open circuit potential (OCP) measurements and potentiodynamic polarization tests were conducted. The results, presented in Figure 9 and Table 3, reveal significant differences in corrosion resistance among the weld metal (W), CGHAZ, FGHAZ, and base metal (BM), highlighting the role of electrochemical factors in crack initiation and propagation.

The OCP results (Figure 9a) indicate that the weld metal exhibited the mostnoble potential, signifying superior corrosion resistance. In contrast, the CGHAZ and FGHAZ exhibited relatively lower potentials, comparable to the base metal. This suggests that the CGHAZ and FGHAZ are more electrochemically active, making them preferential sites for localized anodic dissolution. Potentiodynamic polarization tests (Figure 9b) further confirm this trend. The weld metal displayed the lowest corrosion current density (Icorr = 3.83 × 10^−7^ A/cm^2^), whereas the CGHAZ and FGHAZ exhibited significantly higher values (Icorr = 1.99 × 10^−6^ A/cm^2^ and 4.99 × 10^−6^ A/cm^2^, respectively), reinforcing their susceptibility to corrosion-driven material loss. The base metal exhibited the highest corrosion current (Icorr = 9.00 × 10^−6^ A/cm^2^), indicating a lower overall corrosion resistance compared to the weld metal.

To further quantify the corrosion resistance of each region, the polarization resistance (R_p_) was calculated using the Stern–Geary equation based on the measured Tafel slopes and corrosion current densities. The results showed that the weld metal exhibited the highest R_p_ value (7034 Ω·cm^2^), followed by the CGHAZ (5126 Ω·cm^2^), FGHAZ (3535 Ω·cm^2^), and base metal (2618 Ω·cm^2^). These values support the conclusion that the weld metal is the most corrosion-resistant region, while the CGHAZ and FGHAZ are more susceptible to localized electrochemical degradation due to their lower polarization resistance and microstructural instability.

However, the CGHAZ and FGHAZ remain the most critical regions for localized corrosion-related degradation. Despite the higher Icorr value of the BM, its uniform microstructure distributes corrosion more homogeneously, leading to general corrosion rather than localized attack. In contrast, the CGHAZ and FGHAZ, which exhibit significant microstructural heterogeneity and strain localization, are more prone to preferential dissolution at stress-concentrated sites. This distinction suggests that corrosion-driven material loss in the CGHAZ and FGHAZ had a greater influence on crack propagation than the uniform corrosion observed in the BM.

The electrochemical results strongly correlate with the mechanical and microstructural findings. The increased anodic dissolution tendency of the CGHAZ and FGHAZ, combined with their inherent strain localization and residual stress concentration, provides a favorable condition for crack tip sharpening. The preferential dissolution of less corrosion-resistant phases in these regions could have further intensified the crack growth process, reducing the mechanical threshold required for propagation under thermal and mechanical loading conditions [20].

These findings suggest that the observed crack extension along the weld toe was not solely due to mechanical loading. Instead, it resulted from coupled mechanical and electrochemical degradation. Microstructural heterogeneity affected not only the mechanical integrity but also promoted localized corrosion. The critical role of corrosion-assisted crack propagation further underscores the necessity of considering electrochemical factors when assessing failure mechanisms in welded structures.

### 3.6. Corrosion Failure Mechanisms

In an aqueous environment, low-carbon steel materials such as the pipe used in this study are prone to corrosion pitting due to their relatively low corrosion resistance. Figure 4 and Figure 5 provide cross-sectional microstructural images and hardness test results, respectively, where pits can be seen along the base metal surface. However, it should be noted that not all pits directly corresponded to the crack initiation sites. This indicates that pitting corrosion alone is insufficient to cause failure. Instead, it is presumed that crack initiation and propagation were the result of a combination of factors such as microstructural defects, internal/external stress, and local mechanical degradation. These complex interactions may have gradually intensified localized damage, ultimately leading to failure. Therefore, the failure mechanisms observed in the weldment indicate a strong interrelation between microstructural characteristics, mechanical properties, and corrosion resistance, particularly in the formation and propagation of sharp crack tips. The combined effects of localized stress concentration and electrochemical activity at the crack tip played a crucial role in accelerating crack propagation along the weld toe. The stress sensitivity of the crack tip is significantly influenced by its sharpness, as a highly localized stress field develops around an acutely pointed defect. In the present study, the CGHAZ and FGHAZ exhibited higher residual stresses and lower corrosion resistance than the weld metal (W). The increased anodic dissolution tendency in these regions, as demonstrated by their lower OCV values and higher corrosion current densities (Icorr), suggests that localized material loss could have intensified stress concentration effects at the crack tip. This phenomenon is critical in governing crack propagation, as the dissolution of material at the crack front reduces the effective crack tip radius, thereby further amplifying the local stress field. From a mechanical standpoint, the stress concentration at the crack tip is described by the Inglis equation for an elliptical flaw under far-field stress.

(1) $$\sigma_{max}=\sigma_{\mathrm{applied}}\left(1+2\sqrt{\frac{a}{r}}\right),$$

where σ*_max_* represents the maximum local stress at the crack tip, σ*_applied_* is the applied stress, *a* is the crack length, and *r* is the crack tip radius.

This relationship illustrates that as the crack tip becomes sharper, the local stress at the crack tip increases dramatically, making the defect more prone to propagation. Given that CGHAZ and FGHAZ exhibit high residual stress and strain localization, these regions inherently amplify the stress sensitivity of crack tips. Additionally, EBSD analysis indicated that CGHAZ and FGHAZ exhibited higher KAM values, suggesting an increased density of geometrically necessary dislocations (GNDs). The accumulation of GNDs leads to localized strain concentration, further elevating the susceptibility of these regions to crack initiation. The combined effects of high stress concentration, electrochemical degradation, and thermal exposure reinforce the likelihood of stress corrosion-assisted crack growth in these regions [21,22,23,24].

These findings highlight the critical role of stress–corrosion coupling in crack propagation, where the synergy between mechanical stress concentration and electrochemical degradation governs failure progression. The enhanced crack tip sharpness due to corrosion-driven material loss accelerates local stress intensification, ultimately reducing the mechanical threshold for crack growth. This underscores the necessity of mitigating both residual stresses and corrosion susceptibility in welded structures, particularly in the HAZ, to enhance service life and structural integrity.

## 4. Conclusions

This study investigated the failure mechanism of a 750A dual-insulated pipeline welded joint, with a primary focus on crack initiation and propagation in the heat-affected zone (HAZ). A comprehensive analysis, including mechanical stress evaluation, microstructural characterization, and electrochemical testing, was conducted to establish the root cause of failure. The main findings are summarized below.

1. Microstructural analysis revealed that coarse-grained HAZ (CGHAZ) contained coarse grains and exhibited the highest hardness (>300 HV), indicating increased brittleness. KAM analysis showed a high density of geometrically necessary dislocations (GNDs), leading to localized strain and greater susceptibility to crack initiation.
2. Finite element analysis confirmed that CGHAZ experienced the highest stress concentration, with peak localized stresses exceeding 475 MPa. This stress level surpassed the yield strength of the base metal (349 MPa) and approached that of the weld metal (384 MPa), indicating that CGHAZ was subjected to severe stress conditions. Thermal expansion and residual welding stresses created a localized stress gradient at the weld toe and CGHAZ boundary, further intensifying crack susceptibility.
3. Electrochemical tests confirmed that the HAZ exhibited low open circuit potential (OCP), acting as the anodic region and promoting localized corrosion. The presence of micro-pitting and oxide penetration along the fracture surface further indicates that electrochemical degradation, in combination with operating and residual stress, contributed to the initiation and propagation of stress corrosion cracking (SCC).

## Figures and Tables

**Table 1:** Chemical composition of a pipe [unit: wt.%].

C	Si	Mn	S	P	Fe
0.30	0.35	0.30–1.00	0.040	0.040	Bal.

**Table 2:** A summary of the tensile test result.

Mechanical Properties	Yield Strength (MPa)	Tensile Strength (MPa)	Elongation (%)
Base metal (BM)	348.6	505.9	41.2
Welded part (W)	384.1	531.6	18.7

**Table 3:** Potential dynamic curve result data.

Microstructure	E_corr_ (mV)	I_corr_ (A/cm^2^)	β_a_ (mV)	β_c_ (mV)	R_p_ (Ω·cm^2^)
W	−137	3.83 × 10^−7^	6.4	203	7034
CGHAZ	−402	1.99 × 10^−6^	38.1	62.1	5126
FGHAZ	−408	4.99 × 10^−6^	63.2	114	3535
BM	−388	9.00 × 10^−6^	72.1	219	2618

## Data Availability

The original contributions presented in this study are included in the article. Further inquiries can be directed to the corresponding author.

## References

[1] McCafferty E. (2010). Introduction to Corrosion Science.

[2] Song M.J., Choi G., Chae H., Kim W.C., Kim H., Kim J.G., Lee S.Y. (2021). Corrosion failure analysis of flow plate in plate heat exchanger. Corros. Sci. Technol..

[3] Kim H.T., Kil S.C., Hwang W.S., Cho W.S. (2007). Investigation on the corrosion behaviour of weld structure. Corros. Sci. Technol..

[4] Song M.J., Choi G., Kim W.C., Lee S.Y. (2022). Cause of Corrosion and Evaluation of Material Corrosion Resistance on Underground Heat Transport Facilities Connected to Manhole. J. Korean Soc. Heat Treat..

[5] Cho J., Chae H., Kim H., Kim J.G., Kim W.C., Lee S.Y. (2022). Failure Analysis of Stress Reliever in Heat-Transport Pipe of District Heating System. Corros. Sci. Technol..

[6] Lins V.F.C., Guimaraes E.M. (2007). Failure of a heat exchanger generated by an excess of SO_2_ and H_2_S in the sulfur recovery unit of a petroleum refinery. J. Loss Prev. Process Ind..

[7] Tang Z., Wang Z., Lu Y., Sun P. (2021). Cause analysis and preventive measures of pipeline corrosion and leakage accident in alkylation unit. Eng. Fail. Anal..

[8] Song M.J., Kim W.C., Kim H., Kim J.G., Lee S.Y. (2023). Corrosion Failure Analysis of a Biogas Pipe. J. Korean Soc. Heat Treat..

[9] Kim S.H., Lee S.M., Lee J.H. (2024). A two-phase flow accelerated corrosion study on water wall tube of coal-fired boiler according to flexible operation. Corros. Sci. Technol..

[10] Bain D.I., Christophersen D.L. (2003). Some common mechanisms leading to failures in heat recovery steam generators. Proceedings of the NACE CORROSION Conference.

[11] Dooley B., Anderson B. (2009). HRSG assessments identify trends in cycle chemistry thermal transient performance. Power Plant Chem..

[12] Li Q., Yao Q., Sun L., Ma H., Zhang C., Wang N. (2023). Effect of micro-galvanic corrosion on corrosion fatigue cracking of the weld joint of high strength bridge steel. Int. J. Fatigue.

[13] Huang Y., Zhang J., Xuan F.Z., Ma Y. (2024). Modeling and prediction of galvanic corrosion for an overlaying welded structure. J. Mater. Eng. Perform..

[14] Lin M.B., Gao K., Wang C.J., Volinsky A.A. (2012). Failure analysis of the oil transport spiral welded pipe. Eng. Fail. Anal..

[15] Xia Z., Huang Y., Zhong J., Guan K. (2023). Cracking failure analysis on a high-frequency electric resistance welding pipe in buried fire water pipeline. Eng. Fail. Anal..

[16] Ul-Hamid A., Tawancy H.M., Abbas N.M. (2005). Failure of weld joints between carbon steel pipe and 304 stainless steel elbows. Eng. Fail. Anal..

[17] Shirinzadeh-Dastgiri M., Mohammadi J., Behnamian Y., Eghlimi A., Mostafaei A. (2015). Metallurgical investigations and corrosion behavior of failed weld joint in AISI 1518 low carbon steel pipeline. Eng. Fail. Anal..

[18] Gadala M., Gadala I., Gomaa A. (2025). Failure assessment of seam-welded pipe under fatigue and thermal loading. Eng. Fail. Anal..

[19] Li Y., Hu T., Li Q., Wu Y., Wang L., You Y., Wang B. (2024). Evaluation of the stress corrosion crack growth behaviour of high-strength marine steel based on model of crack tip mechano-electrochemical effect. J. Mater. Sci. Technol..

[20] Winzer N., Atrens A., Song G., Ghali E., Dietzel W., Kainer K.U., Blawert C. (2005). A critical review of the stress corrosion cracking (SCC) of magnesium alloys. Adv. Eng. Mater..

[21] Saravanan N., Karamched P.S., Liu J., Rainasse C., Scenini F., Lozano-Perez S. (2020). Using local GND density to study SCC initiation. Ultramicroscopy.

[22] Meisnar M., Vilalta-Clemente A., Moody M., Arioka K., Lozano-Perez S. (2016). A mechanistic study of the temperature dependence of the stress corrosion crack growth rate in SUS316 stainless steels exposed to PWR primary water. Acta Mater..

[23] Kim Y.S., Nam H.S., Kwon Y.H., Kim S.W., Kim H.P., Chang H.Y. (2010). Relationship between the initiation and propagation of SCC and the electrochemical noise of Alloy 600 for the steam generator tubing of nuclear power plants. Corros. Sci. Technol..

[24] Borchert M., Mori G., Bischof M., Tomandl A. (2015). Accelerated SCC testing of stainless steels according to corrosion resistance classes. Corros. Sci. Technol..

